# Risikofaktoren und Behandlung des Postpunktionskopfschmerzes – Analyse der deutschen Patientendaten der internationalen EPIMAP-Studie

**DOI:** 10.1007/s00101-025-01540-7

**Published:** 2025-05-21

**Authors:** S. Kroegel, C. von Heymann, A. Schyns-van den Berg, K. Becke, P. Kranke, H. Lewald, S. Müller, E. Muggleton, C. Neumann, H. Ohnesorge, S. Piper, L. Kaufner

**Affiliations:** 1 Klinik für Anästhesiologie und Intensivmedizin CCM/CVK, Campus Virchow-Klinikum, Charité – Universitätsmedizin Berlin, Berlin, Deutschland; 2 Klinik für Anästhesie, Intensivmedizin, Notfallmedizin und Schmerztherapie, Vivantes Klinikum im Friedrichshain, Berlin, Deutschland; 3 Department of Anesthesiology, Albert Schweitzer Hospital, Dordrecht, Niederlande; 4 Department of Anesthesiology, Leiden University Medical Centre, Leiden, Niederlande; 5 Abteilung für Anästhesie und Intensivmedizin, Klinik Hallerwiese-Cnopfsche Kinderklinik, DIAKONEO KdöR, Nürnberg, Deutschland; 6 Klinik und Poliklinik für Anästhesiologie, Intensivmedizin, Notfallmedizin und Schmerztherapie, Universitätsklinikum Würzburg, Würzburg, Deutschland; 7 Klinik für Anästhesie und Intensivmedizin, Klinikum rechts der Isar, München, Deutschland; 8 Klinik für Anästhesie, Notfall- und Schmerzmedizin, Gemeinschaftsklinikum Mittelrhein, Koblenz, Deutschland; 9 Abteilung für Anästhesie, Rotkreuz-Klinikum, München, Deutschland; 10 Klinik für Anästhesiologie und Operative Intensivmedizin, Universitätsklinikum Bonn, Bonn, Deutschland; 11 Klinik für Anästhesiologie und Operative Intensivmedizin, Universitätsklinikum Schleswig-Holstein, Kiel, Deutschland; 12 Institut für Medizinische Informatik und Institut für Biometrie und klinische Epidemiologie, Charité – Universitätsmedizin Berlin, Berlin, Deutschland

**Keywords:** Risikofaktoren, Duraperforation, Periduralanästhesie, Blut-Patch, Konservative Therapie, Risk factors, Dural puncture, Epidural anesthesia, Blood patch, Conservative treatment

## Abstract

**Hintergrund:**

Die akzidentelle Duraperforation (ADP) ist eine seltene, aber ernst zu nehmende Komplikation der rückenmarknahen Anästhesie, die zu einem Postpunktionskopfschmerz (PDPH), welcher das Wohlbefinden der betroffenen Frauen erheblich beeinträchtigt, führen kann.

**Ziel der Arbeit:**

Die vorliegende Arbeit untersucht die Patientinnencharakteristika, die Behandlung und das Behandlungsergebnis des PDPH nach ADP in der Geburtsmedizin und vergleicht eine konservative Therapie mit einem epiduralen Blut-Patch (EBP). Es werden charakteristische Merkmale, Symptomverläufe, Nebenwirkungen und Komplikationen beider Therapieansätze dargestellt.

**Methoden:**

Es wurden Daten von 73 Patientinnen aus 9 deutschen Studienzentren der europäischen Studie European Practices in the Management of Accidental Dural Puncture in Obstetrics (EPIMAP), einer prospektiven multizentrischen Kohortenstudie, deskriptiv analysiert.

**Ergebnisse:**

Im Zeitraum von 2016 bis 2018 erhielten 56 der 73 (76,7 %) eingeschlossenen Patientinnen einen EBP, 17 Patientinnen wurden konservativ behandelt (23,3 %). Im Kollektiv mit der konservativen Therapie wurde auf der numerischen Schmerzskala (NRS) mit Interquartilenbereich (IQR) im Liegen ein medianes Schmerzniveau von 2 (IQR 1; 3) und im Stehen von 7 (5; 8) angegeben. Die andere Gruppe gab vor Anlage des EBP eine Schmerzintensität im Liegen von NRS 3 (1; 7) und im Stehen von NRS 8,5 (7; 10) an. Das Schmerzniveau 24 h nach EBP lag bei einem NRS von 0 (0; 2) im Liegen und von 1 (0; 2) im Stehen. Die konservativ behandelten Patientinnen beschrieben bei Entlassung einen Schmerzrückgang im Liegen auf NRS 0 (0; 1) und im Stehen auf NRS 2 (1; 4). 15 Patientinnen wurden nach einem EBP mit erneutem PDPH stationär aufgenommen (26,8 %), 9 erhielten einen zweiten EBP (60 %).

**Diskussion:**

Der EBP stellt eine effektive Behandlungsoption zur Senkung der Schmerzintensität eines Postpunktionskopfschmerzes dar, wobei die Wahl zwischen EBP und konservativer Therapie v. a. von der Intensität des Kopfschmerzes beeinflusst zu sein scheint. Die Ergebnisse dieser Untersuchung unterstreichen die Bedeutung eines individualisierten Behandlungsansatzes.

## Abstract (en)

**Background:**

Accidental dural puncture (ADP) is a rare but serious complication that can lead to post-dural puncture headache (PDPH), a condition known to significantly impair the well-being of affected women.

**Objectives:**

The present study examines the patient characteristics, symptoms, management and outcomes of women with PDPH after ADP and focuses on differences between conservative management and epidural blood patch (EBP).

**Materials and methods:**

The data of nine German study sites of the European prospective, multicenter cohort EPIMAP (European Practice in the Management of Accidental Dural Puncture in Obstetrics) trial were analyzed. Only descriptive analyses were performed due to the small sample size.

**Results:**

Data were collected between 2016 and 2018 and 73 patients were included in Germany. Of the 73 patients (76.7%), 56 received an EBP and 17 were treated conservatively (23.3%). In the conservative treatment group, the median numeric rating score of pain (NRS) plus interquartile range (IQR) during the hospital stay was 2 (1; 3) at rest and 7 (5; 8) while sitting or standing. Upon discharge, the NRS had decreased to 0 (0; 1) at rest and 2 (1; 4) while sitting or standing. In the EBP group NRS was 3 (1; 7) at rest and 8.5 (7; 10) while sitting or standing 6–12 h before the intervention. Furthermore, 24 h after the EBP, NRS was 0 (0; 2) at rest and 1 (0; 2) while sitting or standing. A total of 15 women were readmitted to the hospital for recurrent PDPH after EBP (26.8%). A second EBP was administered to 9 patients (60%).

**Conclusion:**

The EBP is an effective treatment option for post-puncture dural headache. The choice between the EBP and a conservative treatment seems to be influenced by the intensity of the PDPH. These results underline the importance of an individual treatment approach in patients with PDPH.

## Einleitung

Aufgrund des weit verbreiteten Einsatzes von rückenmarknahen Verfahren zur Erleichterung des Geburtsschmerzes stellen akzidentelle Durapunktionen (ADP) in der geburtshilflichen Anästhesie eine seltene, aber mit erheblichen Beschwerden verbundene Komplikation dar: Sie treten in 0,3–1,5 % aller Epiduralanästhesien auf, und 50–88 % der betroffenen Frauen leiden im Anschluss an Symptomen eines Postpunktionskopfschmerzes („post-dural puncture headache“, PDPH) [3, 7].

Laut der Internationalen Kopfschmerzgesellschaft ist PDPH definiert als Kopfschmerz, der innerhalb von 5 Tagen nach einer Durapunktion auftritt und sich als dumpfer, frontaler Schmerz mit Ausstrahlung nach okzipital präsentiert [15]. Charakteristisch sind eine Zunahme des Schmerzes innerhalb von 15 min beim Sitzen oder im Stehen und eine Besserung der Symptomatik im Liegen. Der Kopfschmerz kann mit Nackensteifigkeit, Schwindel, Lichtscheu, akustischen (Tinnitus) und optischen Symptomen (Doppelbilder) sowie Übelkeit und Erbrechen [3] assoziiert sein.

Der Pathomechanismus wird in einem anhaltenden Austritt von Liquor über den Stichkanal in der Dura gesehen. Diese Leckage führt zu einer Reduktion des Liquorvolumens und dadurch zu einem Unterdruck im Subarachnoidalraum mit Zug auf die schmerzsensiblen Hirngefäße, Meningen und Hirnnerven [25]. Die vielfältigen klinischen Manifestationen des PDPH machen deutlich, wie stark die betroffenen Frauen in den ersten Tagen nach der Geburt beeinträchtigt sein können, sodass eine adäquate Versorgung der Neugeborenen teilweise nicht möglich ist. Darüber hinaus kann der PDPH zu einem verlängerten Aufenthalt und zur Wiederaufnahme ins Krankenhaus führen sowie mit einem erhöhten Risiko für weitere neurologische Folgekomplikationen, z. B. Subduralhämatomen oder chronischen Schmerzsyndromen, einhergehen [9].

Aufgrund der Vielzahl von betroffenen Frauen sind wirksame Therapiestrategien nötig: Das konservative Management umfasst u. a. Bettruhe und die medikamentöse Therapie mit peripher wirksamen Analgetika und Koffein. Darüber hinaus kann ein epiduraler Blut-Patch (EBP) zum Einsatz kommen; dieser scheint allen weiteren interventionellen Therapieformen (z. B. Ganglion-sphenopalatinum-Blockade) überlegen zu sein [8]. Beim EBP werden je nach Anwender bis zu 30 ml autologen Blutes in den Epiduralraum auf Höhe der Duraverletzung injiziert, welches koaguliert und so das Duraleck verschließen und den Unterdruck aufheben soll [12, 26, 27]. Der EBP zeigt hohe Erfolgsraten, geht jedoch auch mit dem Risiko von Infektionen oder erneuter Durapunktion sowie Nebenwirkungen wie Rückenschmerzen, Parästhesien oder Therapieversagen einher. Dies macht systematische und randomisierte Studien nötig, mit deren Hilfe Technik, Sicherheit und Benefits des EBP beschrieben und mit dem konservativen Management verglichen werden können.

Ziel dieser Arbeit ist es, die charakteristischen Merkmale, den Symptomverlauf sowie Erfolgsraten beider Therapieansätze aufzuzeigen und zu beschreiben, welche Faktoren die Wahl des Behandlungsverfahrens beeinflusst haben könnten. Hierfür wurden die Daten der deutschen Studienzentren der Studie European Practices in the Management of Accidental Dural Puncture in Obstetrics (EPIMAP) analysiert [13, 14].

## Methoden

Bei der EPIMAP-Studie (clinicaltrials.gov, NCT 023362828) handelt sich um eine prospektive, multizentrische, internationale Kohortenstudie, an der 158 Zentren, davon 9 deutsche Geburtskliniken, teilnahmen. Die Ethikvoten wurden über die Ethikkommissionen der teilnehmenden Kliniken eingeholt.

Die Methodik der EPIMAP-Studie wurde in 2 Vorpuplikationen beschrieben und hier kurz zusammengefasst [13, 14]. Eingeschlossen wurden alle Patientinnen im Alter von 18+ Jahren, die während der Wehentätigkeit eine Epiduralanästhesie erhielten, wenn eine ADP vermutet/bestätigt (Liquor sichtbar in der Epiduralnadel) und die Diagnose PDPH postpartum gestellt wurde, sofern sie einer Teilnahme schriftlich zugestimmt hatten.

Ausschlusskriterien waren Kliniken mit weniger als 500 Geburten/Jahr, Patientinnen mit einem PDPH nach einer Spinalanästhesie, der fehlende sichere Nachweis einer ADP bei Anlage einer CSE, Sprachbarriere, Vorerkrankungen, welche die Einhaltung des Studienprotokolls erschweren, sowie Patientinnen, die sich erst 5 Tage nach der PDA/CSE mit PDPH-Symptomen vorstellen.

Bei Patientinnen mit charakteristischer Symptomatik, bei denen eine ADP vermutet wurde oder nachweislich stattgefunden hatte, erfolgte die Untersuchung durch Fachärzt:innen für Anästhesiologie und die Bestätigung der Diagnose. Die Kopfschmerzintensität wurde mittels einer numerischen Rating-Skala (NRS) quantifiziert, in der „0“ für völlige Schmerzfreiheit und „10“ für den stärksten vorstellbaren Schmerz stehen. Die eingeschlossenen Patientinnen wurden bis zur Entlassung aus dem Krankenhaus und nach 3 Monaten telefonisch nachbefragt. Jede Wiederaufnahme, die auf den PDPH oder den EBP zurückzuführen war, und der Verlauf des Kopfschmerzes wurden aufgezeichnet.

Alle beschriebenen Untersuchungen an den Patientinnen wurden mit Zustimmung der zuständigen Ethikkommission, im Einklang mit nationalem Recht sowie gemäß der Deklaration von Helsinki von 1975 (in der aktuellen, überarbeiteten Fassung) durchgeführt. Die Rohdaten der hier analysierten Patientinnen können auf Anfrage eingesehen werden.

### Definitionen

ADP wurde definiert als sichtbarer Liquor in der Epiduralnadel oder als ein positiver Aspirationstest über den Epiduralkatheter oder das Auftreten einer Spinalanästhesie nach Injektion des Lokalanästhetikums in den Epiduralkatheter.

Der PDPH wurde nach den Vorgaben der Internationalen Kopfschmerzgesellschaft wie folgt definiert [15]:

1. Kopfschmerz, der sich innerhalb von 15 min nach dem Aufsetzen oder Hinstellen verschlimmert und sich innerhalb von 15 min nach dem Hinlegen verbessert;
2. Kopfschmerz, der sich innerhalb von 5 Tagen nach einer Durapunktion entwickelt;
3. Kopfschmerz, der begleitet sein kann von Nackensteifigkeit, Schwindel, Seh- oder Hörstörungen.

Chronischer Rücken- oder Kopfschmerz wurde definiert als NRS ≥ 3 nach 3 Monaten. Spontane Besserung wurde definiert als NRS < 3 im Sitzen und im Stehen innerhalb von 24 h nach der PDPH-Diagnose.

Die Studie wurde von der European Society of Anaesthesiolgy and Intensive Care (ESAIC) gesponsort und koordiniert. Die Daten wurden nach Protokoll, Good Clinical Practice und den länderspezifischen gesetzlichen Vorschriften erhoben, dokumentiert und ausgewertet.

*Statistik*: Der ungepaarte *t*-Test wurde zur statistischen Analyse bei normal verteilten Variablen, der Mann-Whitney-Test bei nicht normal verteilten Daten angewendet. Die statistische Analyse erfolgte mit SPSS-Version 29, IBM Deutschland GmbH, Böblingen.

Die Ergebnisse sind als absolute Zahlen und in Prozent, die Schmerzangaben auf der NRS-Skala werden als Mediane mit Interquartilbereich (IQR) angegeben. An dieser Stelle sei darauf hingewiesen, dass es sich aufgrund der kleinen Fallzahl um eine beschreibende und explorative Analyse handelt.

## Ergebnisse

### Patientinneneigenschaften und Merkmale der PDA-Anlage, Diagnosestellung und Behandlung

Die Daten wurde im Zeitraum vom 01.01.2016 bis 31.12.2018 erhoben. Die deutschen Studienzentren schlossen 73 Patientinnen ein, von denen 56 Patientinnen mit einem EBP (EBP-Gruppe) und 17 Patientinnen ohne behandelt (Non-EBP-Gruppe) wurden. Darüber hinaus erhielten 2 Patientinnen weder einen Blut-Patch noch eine konservative Therapie und wurden nicht in unsere Auswertung eingeschlossen.

Tab. 1 zeigt charakteristische Eigenschaften der eingeschlossenen Patientinnen: Betroffene in der Non-EBP-Gruppe wiesen dabei mehr Vorerkrankungen auf und waren häufiger Raucherinnen als die Patientinnen der EBP-Gruppe.

**Tab. 1:** Demografische Eigenschaften der Patientinnen

	Total (*n* = 73)	EBP (*n* = 56)	No-EBP (*n* = 17)
*Mütterliches Alter *(in Jahren), Mittelwert (SD)	32 (± 5)	33 (± 4)	28 (± 5)
*Parität*, Multipara, *n* (%)	28 (38)	21 (38)	7 (41)
*Body-Mass-Index *(BMI) (kg/m^2^), Mittelwert (SD)	27 (± 5)	27 (± 6)	26 (± 4)
*Vorerkrankungen, n* (%)			
Neuroaxiale Anästhesie	16 (22)	12 (21)	4 (24)
PDPH	2 (3)	1 (2)	1 (6)
Chronischer Kopfschmerz	4 (6)	3 (5)	1 (6)
Migräne	15 (21)	10 (18)	5 (29)
Pathologie der Wirbelsäule	7 (10)	3 (5)	4 (24)
Chronische Rückenschmerzen	5 (7)	3 (5)	2 (12)
*Raucher, *ja, *n* (%)	3 (4)	1 (2)	2 (12)
*Beruf, n* (%)			
Verwaltung	10 (14)	6 (11)	4 (24)
Lehrtätigkeit	10 (14)	8 (14)	2 (12)
Gesundheitswesen	13 (18)	10 (18)	3 (18)
Arbeitnehmer (ohne Universitätsausbildung)	7 (10)	5 (9)	2 (12)
Arbeitnehmer (mit Universitätsausbildung)	21 (29)	18 (32)	3 (18)
Kein Beruf	12 (16)	9 (16)	3 (18)
*Höchster Bildungsabschluss, n* (%)			
Realschulabschluss	12 (16)	9 (16)	3 (18)
Abitur	21 (29)	14 (25)	7 (41)
Universitätsabschluss	39 (54)	32 (57)	7 (41)
*Geburtsmodus, n* (%)			
Spontangeburt	43 (59)	33 (59)	10 (59)
Instrumentierte Entbindung	5 (7)	5 (9)	0 (0)
Kaiserschnitt	25 (34)	18 (32)	7 (41)

Ergebnisse werden als Mittelwert und Standardabweichung dargestellt

*EBP* epiduraler Blut-Patch; *PDPH* „post-dural puncture headache“ (Postpunktionskopfschmerz); *SD* Standardabweichung

Die Charakteristika der Anlage der Epiduralanästhesie sowie die Diagnose- und Behandlungskriterien sind in Tab. 2 zusammengefasst: Bei rund 80 % der Patientinnen wurde in beiden Gruppen eine 18-G-Nadel zur Periduralkatheter(PDK)-Anlage sowie Kochsalzlösung als Medium verwendet. Die sitzende Position bei der PDK-Anlage war in der EBP-Gruppe häufiger, über die Hälfte der Frauen wurden dabei auf Höhe L3/4 punktiert, ein Drittel der Betroffenen erhielt die Epiduralanästhesie in Höhe L2/3. In der Non-EBP-Gruppe war die Epiduralanästhesie überwiegend in Höhe L3/4 durchgeführt worden. Die Anzahl der Patientinnen mit schwieriger PDK-Anlage war bei beiden Gruppen nahezu identisch, ebenso die Berufserfahrung des Punktierenden. Die EBP-Gruppe zeigte jedoch eine deutlich höhere Rate an multiplen Punktionsversuchen. Auch wurde die Diagnose in der EBP-Gruppe im Mittel 8 h früher gestellt als bei den konservativ behandelten Patientinnen. Der PDPH trat hauptsächlich okzipital sowie im Nackenbereich auf. Darüber hinaus litten Betroffene aus der EBP-Gruppe am häufigsten an auditorischen Symptomen, während die Non-EBP-Gruppe vermehrt Schwindel angab. Stillen war bei den konservativ behandelten Müttern ohne Einschränkung möglich, hingegen waren es im EBP-Kollektiv 10 %, die vor der Anlage des EBP nicht in der Lage waren, ihr Kind selbst zu stillen. Die konservativen Therapiemöglichkeiten wie beispielsweise Bettruhe und Schmerzmittelgaben oder die Anwendung von Koffeintabletten wurden in der EBP-Gruppe vor Anlage des EBP im Vergleich mit der Non-EBP-Gruppe stärker genutzt (Tab. 1).

**Tab. 2:** Charakteristika von Diagnosestellung, PDPH-Management und Epiduralkatheteranlage

	Gesamt (*n* = 73)	EBP (*n* = 56)	No-EBP (*n* = 17)
*Nadelgröße, n* (%)			
16 G	10 (14)	8 (14)	2 (12)
17 G	4 (5)	3 (5)	1 (6)
18 G	59 (81)	45 (81)	14 (82)
*Mittel zur Ermittlung des LOR, n* (%)			
Kochsalzlösung	71 (97)	54 (96)	17 (100)
Luft	2 (3)	2 (4)	0 (0)
*Position der Patientin während PDA, n* (%)			
Liegend	7 (10)	3 (5)	4 (24)
Sitzend	66 (90)	53 (95)	13 (76)
*Höhe der PDA, n* (%)			
L1–2	3 (4)	3 (5)	0 (0)
L2–3	22 (30)	18 (32)	4 (26)
L3–4	42 (58)	30 (54)	12 (71)
L4–5	6 (8)	5 (9)	1 (6)
*Schwierige Punktion, n* (%)	28 (38)	22 (39)	6 (35)
*Multiple Punktionsversuche, n* (%)	45 (62)	40 (71)	5 (29)
*Dauer* (h), Median (Q1/Q3)			
PDK-Anlage bis PDPH-Diagnose	41 (26/55)	49 (31/71)	40 (22/53)
PDK-Anlage bis EBP	Na	72 (48/97)	Na
*Intrathekaler Katheter nach ADP, n* (%)	3 (4)	2 (4)	1 (6)
*Erfahrung des/der Ausführenden, n* (%)			
< 6 Monate	1 (1)	1 (2)	0 (0)
6–12 Monate	2 (3)	2 (4)	0 (0)
1–5 Jahre	26 (36)	20 (35)	6 (35)
> 5 Jahre	44 (60)	33 (59)	11 (65)
*Wie wurde der ADP festgestellt? n* (%)			
CSF in Epiduralnadel	32 (44)	25 (45)	7 (41)
CSF im Katheter oder positiver Aspirationstest	6 (8)	5 (9)	1 (6)
Spinalanästhesie nach Testdosis	3 (4)	2 (4)	1 (6)
Typische PDPH-Symptome postpartal	43 (59)	33 (59)	10 (59)
*Weitere Symptome, n* (%)			
Übelkeit und/oder Erbrechen	19 (26)	14 (25)	5 (29)
Auditive Symptome	18 (25)	16 (29)	2 (12)
Doppelbilder	0 (0)	0 (0)	0 (0)
Schwindel	23 (32)	15 (27)	8 (47)
Andere visuelle Symptome	8 (11)	6 (11)	2 (12)
Tinnitus	7 (10)	5 (9)	2 (12)
Andere	11 (15)	9 (16)	2 (12)
*Entlassung vor Symptombeginn, n* (%)	5 (7)	4 (7)	1 (6)
*Stillen trotz PDPH, n* (%)	67 (92)	50 (89)	17 (100)
*Kopfschmerzlokalisation, n* (%)			
Temporal	16 (22)	12 (21)	4 (24)
Okzipital	49 (67)	36 (64)	13 (76)
Frontal	38 (52)	31 (55)	7 (41)
Hals	51 (70)	39 (70)	12 (71)
Schulter	21 (29)	18 (32)	3 (18)
Andere	5 (7)	4 (7)	1 (6)
*Art der Behandlung vor Diagnosestellung, n* (%)			
PCM	42 (58)	35 (63)	7 (41)
NSAID	43 (59)	37 (66)	6 (35)
Koffein	12 (15)	12 (21)	0 (0)
Opioide	3 (4)	3 (5)	0 (0)
Flüssigkeit	33 (45)	27 (48)	6 (35)
Bettruhe	20 (27)	18 (32)	2 (12)

Ergebnisse sind als Mittelwert und Standardabweichung sowie als Median und Interquartilabstand dargestellt

*ADP* akzidentelle Durapunktion; *EBP* epiduraler Blut-Patch; *PDPH* „post-dural puncture headache“ (Postpunktionskopfschmerz); *CSF* „cerebrospinal fluid“ (Liquor); *PCM* Paracetamol; *NSAID* „non-steroidal anti-inflammatory drug“ (nichtsteroidale Antirheumatika); *Q1* 1. Quartil; *Q3* 3. Quartil; *na* keine Angabe; *PDK* Periduralkatheter

### Behandlung des PDPH nach der Diagnosestellung

Die mediane Zeit zwischen der PDK-Anlage und Diagnose eines PDPH lag in der Non-EBP-Gruppe bei 51 h, in der EBP-Gruppe bei 43 h. Letztere erhielt im Median 92 h nach PDK-Anlage den EBP. Wie in Tab. 2 zu sehen, kamen konservativen Maßnahmen in beiden Gruppen zur Anwendung, wobei jedoch die Patientinnen ohne EBP nicht auf Koffeintabletten oder Opiate zurückgriffen. Patientinnen, die einen EBP erhielten, wurden v. a. mit Paracetamol (35 von 56 Frauen, 62,5 %) und nichtsteroidalen Antirheumatika (NSAID) (37 von 56 Frauen, 66,1 %) behandelt. Von den insgesamt 56 Frauen mit einem EBP wurden 15 (26,8 %) aufgrund von anhaltenden oder wiederaufgetretenen Beschwerden erneut stationär aufgenommen, 9 dieser 15 Patientinnen (60 %) erhielten einen zweiten EBP, bei einer Patientin (6,7 %) wurde wegen ausbleibender Symptomlinderung ein dritter EBP angelegt.

### Nadelgröße

Je größer der Durchmesser der Nadel, desto höher das mediane Schmerzniveau auf der NRS in den ersten 24 h nach der Diagnosestellung im Liegen (16 G: NRS 5,5 (3; 7), 17 G: NRS 3 (2; 4), 18 G: NRS 2 (1; 3)). Im Stehen gleicht sich das Schmerzniveau in etwa an (16 G: NRS 7,5 (6; 10) vs. 17 G NRS 9 (7; 10) vs. 18 G NRS 8 (7; 9)).

### Kopfschmerzintensität und zeitlicher Verlauf

Die mediane Schmerzintensität und der zeitliche Verlauf der Kopfschmerzen sind in Tab. 3 dargestellt. Der mediane NRS-Wert lag in der Non-EBP-Gruppe zum Zeitpunkt der PDPH-Diagnose im Liegen bei 3 (1; 5) und im Stehen bei 8 (6; 9). Bei Entlassung aus dem Krankenhaus wiesen die Patientinnen eine Schmerzintensität von 2 (1; 4) im Stehen und 0 (0; 1) im Liegen auf. In der EBP-Gruppe wurde die Kopfschmerzintensität innerhalb der ersten 24 h nach Diagnosestellung unter Belastung mit 8 (7; 10) und im Liegen mit 3 (1; 5) angegeben. In der Non-EBP-Gruppe betrug die Kopfschmerzintensität 6 (5; 8) im Stehen und 1 (0; 2) im Liegen.

**Tab. 3:** Vergleich der Schmerzintensitäten vom Zeitpunkt der PDPH-Diagnose bis zur Entlassung in der EBP- und Non-EBP-Gruppe

	Total	EBP	Non-EBP
NRS im Liegen zum Zeitpunkt der PDPH-Diagnose, Median (Q1/Q3)	2,0 (1/4) *n* = 73	2,0 (1/4) *n* = 56	3,0 (1/5) *n* = 17
NRS im Sitzen/Stehen zum Zeitpunkt der PDPH-Diagnose, Median (Q1/Q3)	8,0 (7/9) *n* = 73	8,0 (7/9) *n* = 56	8,0 (6/9) *n* = 17
Maximaler NRS im Liegen nach bestätigter Diagnose und innerhalb 0–24 h, Median (Q1/Q3)	2,0 (1/5) *n* = 73	3,0 (1/5) *n* = 56	1,0 (0/2) *n* = 17
Maximaler NRS im Sitzen/Stehen nach bestätigter Diagnose und innerhalb 0–24 h, Median (Q1/Q3)	8,0 (7/9) *n* = 73	8,0 (7/10) *n* = 56	6,0 (5/8) *n* = 17
NRS im Liegen während des Krankenhausaufenthalts, Median (Q1/Q3)	3,0 (2/5) *n* = 19	3,5 (2/6) *n* = 6	2,0 (1/3) *n* = 13
NRS im Sitzen/Stehen während des Krankenhausaufenthalts, Median (Q1/Q3)	7,0 (6/9) *n* = 19	8,5 (6/10) *n* = 6	7,0 (5/8) *n* = 13
NRS im Liegen bei Entlassung, Median (Q1/Q3)	0 (0/1) *n* = 20	0 (0/2) *n* = 7	0 (0/1) *n* = 13
NRS im Sitzen/Stehen bei Entlassung, Median (Q1/Q3)	2,5 (1/5) *n* = 20	3,0 (1/5) *n* = 7	2,0 (1/4) *n* = 13
Maximaler NRS im Liegen 6–12 h vor EBP, Median (Q1/Q3)	n. a.	3,0 (1/7) *n* = 56	n. z.
Maximaler NRS im Sitzen/Stehen 6–12 h vor EBP, Median (Q1/Q3)	n. a.	8,5 (7/10) *n* = 56	n. z.
NRS im Liegen nach Applikation des EBP, Median (Q1/Q3)	n. a.	0 (0/2) *n* = 56	n. z.
NRS im Stehen/bei der ersten Mobilisation nach EBP, Median (Q1/Q3)	n. a.	1,0 (0/3) *n* = 55	n. z.
NRS im Liegen 4 h nach EBP, Median (Q1/Q3)	n. a.	0 (0/1) *n* = 55	n. z.
NRS im Stehen 4 h nach EBP, Median (Q1/Q3)	n. a.	1,0 (0/2) *n* = 45	n. z.
NRS im Liegen 24 h nach EBP, Median (Q1/Q3)	n. a.	0 (0/2) *n* = 56	n. z.
NRS im Stehen 24 h nach EBP, Median (Q1/Q3)	n. a.	1,0 (0/2) *n* = 56	n. z.

Ergebnisse sind als Median und Interquartilabstand dargestellt

*EBP* epiduraler Blut-Patch; *NRS* numerische Rating-Skala; *PDPH* „post-dural puncture headache“ (postduraler Punktionskopfschmerz), *Q1* 1. Quartil; *Q3* 3. Quartil; *n.* *a.* nicht angegeben

Das Schmerzmaximum innerhalb von 12 h vor dem EBP wurde unter Belastung mit 8,5 (7; 10) und im Liegen mit 3 (1; 7) angegeben. Nach erfolgreicher Anlage des EBP kam es zu einem Schmerzrückgang im Liegen auf einen medianen NRS-Wert von 0 (0; 2). Zudem war die Intensität des Kopfschmerzes bei der ersten Mobilisation nach EBP-Anlage auf einen NRS-Wert von 1 (0; 2) gesunken. 24 h nach der EBP-Anlage wurde der NRS-Wert im Stehen mit 1 (0; 2) angegeben (Tab. 3).

### Volumen des EBP

In der Gruppe der mit einem EBP behandelten Patientinnen bestehen mit einem Minimum von 8 ml und Maximum von 40 ml große Unterschiede. Eine Korrelation zu Größe oder BMI der Frauen gibt es dabei nicht. Auch das absolute Schmerzmaximum, gemessen mit der NRS, scheint keinen Einfluss auf das Volumen an Blut, das injiziert wurde, zu haben. Lediglich die Erfahrung der Anästhesist:innen lässt einen Zusammenhang vermuten: Kolleg:innen, die mehr als 10 EBP durchgeführt hatten, injizierten häufiger 20–40 ml, während unerfahrenere Ärzt:innen häufiger zwischen 10 und 20 ml injizierten.

### Zweiter Blut-Patch

Aus der EBP-Gruppe (*n* = 56) wurden 15 Patientinnen mit anhaltenden Beschwerden erneut stationär aufgenommen. Von diesen erhielten 9 Frauen einen zweiten EBP. Tab. 4 gibt einen Überblick über die beiden Gruppen und stellt die technischen Details beim ersten im Vergleich zum zweiten EBP sowie die Schmerzintensitäten im zeitlichen Verlauf gegenüber. Auffällig ist, dass die Patientinnen mit einem zweiten EBP, den ersten deutlich früher erhalten hatten – im Median bereits nach 56 h nach der PDK-Anlage – als die Vergleichsgruppe, deren Beschwerden ohne einen zweiten EBP therapiert werden konnten (Median: 88 h). Zudem hatten die Patientinnen mit einem zweiten EBP, den ersten EBP zu fast 50 % im Liegen erhalten. Die Erfahrung der Anästhesist:in spielte dabei keine Rolle, bzw. es waren überwiegend erfahrene Kolleg:innen, die bereits mehr als 10 Blut-Patches durchgeführt hatten, vor Ort. Von den 9 Patientinnen mit einem zweiten EBP gaben 2 weiterhin anhaltende Beschwerden und einen NRS von 10 im Stehen an. Eine Mutter mit einem NRS = 10 nach dem zweiten Blut-Patch erhielt einen dritten Blut-Patch, der zu einer Schmerzreduktion auf einen NRS-Wert = 0 führte (Tab. 4).

**Tab. 4:** Charakteristika der Epiduralanlage, Diagnosestellung und PDPH-Management in Patientinnen mit anhaltendem PDPH nach dem ersten Blut-Patch

	Gesamt (*n* = 15)	Zweiter EBP (*n* = 9)	Kein zweiter EBP (*n* = 6)
*Dauer (h), Median (Q1/Q3)*			
Epiduralanlage bis PDPH-Diagnose	48 (35/60)	45 (38/65)	52 (36/54)
Epiduralanlage bis EBP	67 (53/103)	58 (47/91)	88 (65/105)
*Position der Patientin während Anlage des ersten EBP, n* (%)			
Liegend	4 (27)	4 (44)	0 (0)
Sitzend	11 (73)	5 (58)	6 (100)
*Höhe des ersten EBP, n* (%)			
L1–2	2 (13)	0	2 (33)
L2–3	7 (47)	6 (67)	1 (17)
L3–4	6 (40)	3 (33)	3 (50)
L4–5	0	0	0
*Blutvolumen des ersten EBP in ml, Median (Q1/Q3)*	30 (30/32)	30 (30/30)	34 (31/36)
*Schwierige Punktion beim ersten EBP, n* (%)	0 (0)	0 (0)	0 (0)
*Erfahrung des/der Ausführenden beim ersten EBP, n* (%)			
< 1	0	0	0
1–5	1 (7)	1 (11)	0
5–10	0	0	0
> 10	14 (93)	8 (89)	6 (100)
*Höhe des ersten EBP, n* (%)			
L1–2	n. a.	0	n. a.
L2–3	n. a.	5 (56)	n. a.
L3–4	n. a.	4 (44)	n. a.
L4–5	n. a.	0	n. a.
*Blutvolumen des zweiten EBP in ml, Median (Q1/Q3)*	n. a.	30 (15/34)	n. a.
*Schwierige Punktion beim ersten EBP, n* (%)	n. a.	0	n. a.
*Erfahrung des/der Ausführenden beim zweiten EBP, n *(%)			
< 1	n. a.	1 (11)	n. a.
1–5	n. a.	4 (45)	n. a.
5–10	n. a.	0	n. a.
> 10	n. a.	4 (45)	n. a.
NRS-Wert im Liegen zum Zeitpunkt der PDPH-Diagnose, Median (Q1/Q3)	2 (1/4)	2 (0/3)	3 (2/5)
NRS-Wert im Sitzen/Stehen zum Zeitpunkt der PDPH-Diagnose, Median (Q1/Q3)	8 (7/9)	8 (8/9)	6,5 (6/8)
Maximaler NRS-Wert im Liegen nach bestätigter Diagnose und innerhalb 0–24 h, Median (Q1/Q3)	2 (1/5)	2 (0/7)	3 (2/5)
Maximaler NRS-Wert im Sitzen/Stehen nach bestätigter Diagnose und innerhalb 0–24 h, Median (Q1/Q3)	7 (7/9)	7 (7/8)	7 (6/9)
Maximaler NRS-Wert im Liegen 6–12 h vor erstem EBP, Median (Q1/Q3)	2 (1/5)	1 (0/3)	4,5 (3/7)
Maximaler NRS-Wert im Sitzen/Stehen 6–12 h vor erstem EBP, Median (Q1/Q3)	8 (6/9)	8 (7/9)	6,5 (5/9)
NRS-Wert im Liegen nach Applikation des ersten EBP, Median (Q1/Q3)	0 (0/1)	0 (0/1)	0 (0/1)
NRS-Wert im Stehen/bei der ersten Mobilisation nach erstem EBP, median (Q1/Q3)	1 (0/2)	2 (0/3)	1 (0/1)
NRS-Wert im Liegen 4 h nach erstem EBP, Median (Q1/Q3)	0 (0/1)	0 (0/1)	0 (0/1)
NRS-Wert im Stehen 4 h nach erstem EBP, Median (Q1/Q3)	1 (0/2) *n* = 14	1 (0/2)	1 (0/2) *n* = 5
NRS-Wert im Liegen 24 h nach erstem EBP, Median (Q1/Q3)	0 (0/1)	1 (0/1)	0 (0/1)
NRS-Wert im Stehen 24 h nach erstem EBP, Median (Q1/Q3)	1 (0/2)	1 (0/2)	0,5 (0/1)
NRS-Wert im Liegen 48 h nach erstem EBP, Median (Q1/Q3)	3 (2/8)	7 (3/8)	2 (1/3)
NRS-Wert im Stehen 48 h nach erstem EBP, Median (Q1/Q3)	8 (7/10)	10 (8/10)	6,5 (4/8)
Maximaler NRS-Wert im Liegen 0–12 h vor zweitem EBP, Median (Q1/Q3)	n. a.	4 (3/9)	n. a.
Maximaler NRS-Wert im Sitzen/Stehen 0–12 h vor zweitem EBP, Median (Q1/Q3)	n. a.	10 (8/10)	n. a.
NRS-Wert im Liegen 4 h nach zweitem EBP, Median (Q1/Q3)	n. a.	0 (0/2)	n. a.
NRS-Wert im Stehen 4 h nach zweitem EBP, Median (Q1/Q3)	n. a.	2 (1/5) *n* = 8	n. a.
NRS-Wert im Liegen 24 h nach zweitem EBP, Median (Q1/Q3)	n. a.	0 (0/2)	n. a.
NRS-Wert im Stehen 24 h nach zweitem EBP, Median (Q1/Q3)	n. a.	5 (1/8)	n. a.

Ergebnisse sind als Median und Interquartilabstand dargestellt

*ADP* akzidentelle Durapunktion; *EBP* epiduraler Blut-Patch; *PDPH* „post-dural puncture headache“ (Postpunktionskopfschmerz); *NRS* numerische Rating-Skala; *n.* *a.* nicht angegeben

### Komplikationsmanagement

Insgesamt 8 Patientinnen der EBP-Gruppe unterzogen sich aufgrund anhaltender Beschwerden weiterführender Diagnostik, nahezu alle (7 von 8) erhielten eine Magnetresonanztomographie (MRT). Diese zeigte in 3 Fällen einen Normalbefund, bei 4 Patientinnen konnten bildmorphologisch Zeichen eines Liquorlecks nachgewiesen werden, und in einem Fall wurde eine Abhebung der dorsalen Dura auf Höhe von Th4 bis L2 durch die epidurale Ausbreitung des Blut-Patch von Th3 bis L3 dargestellt.

## Diskussion

Die vorliegende Analyse basiert auf einer prospektiven multizentrischen und europäischen Kohortenstudie (EPIMAP), deren in Deutschland eingeschlossene Patientinnen untersucht wurden. Die wichtigsten Ergebnisse dieser Analyse sind: Die Kopfschmerzintensität (gemessen mit der NRS im Stehen) war bei Patientinnen mit einem EBP höher als in der Gruppe ohne Blut-Patch. Dies galt auch für die Anzahl der frustranen Punktionsversuche, jedoch nicht für die Erfahrung der Behandler. Die verwendeten Nadelgrößen zur epiduralen Punktion waren in beiden Gruppen nahezu identisch. Das injizierte Blutvolumen für den EBP wies eine hohe Streubreite zwischen 8 und 40 ml auf, wobei unerfahrene Behandler häufiger geringere Volumina für den EBP verabreichten. Nach Anlage des EBP berichteten die Patientinnen von einem deutlichen Rückgang der Schmerzintensität um 6 NRS-Punkte im Median. 15 von 56 Patientinnen mit einem EBP wurden aufgrund rekurrenter Schmerzen erneut stationär aufgenommen, von diesen erhielten 9 Frauen einen zweiten EBP und eine Frau einen dritten EBP.

### Risikofaktoren für einen PDPH

Bekannte Risikofaktoren für einen PDPH umfassen junges Alter, weibliches Geschlecht und das Bestehen einer Schwangerschaft. Insbesondere schwangere Frauen sind einem größeren Risiko für PDPH ausgesetzt, da höhere Östrogenlevel den Tonus der zerebralen Gefäße beeinflussen und es demzufolge zu einer stärkeren Gefäßdilatation als Antwort auf den Liquorverlust kommt [1, 17].

Darüber hinaus wird die vaginale Geburt als ein weiterer Risikofaktor postuliert: Durch das Pressen in der Austreibungsphase könnte sich das Duraleck erweitern und mehr Liquor austreten [2].

Nach unseren Daten hatten insgesamt 43 Frauen mit PDPH eine Spontangeburt. Zehn Frauen wurden konservativ behandelt, 33 Patientinnen hingegen erhielten einen EBP. Von den 5 Frauen, die instrumentiert vaginal entbunden wurden, erhielten alle einen EBP. Es wäre denkbar, dass hier der potenziell schwierigere Verlauf der Austreibungsphase für die stärkeren Beschwerden und die Anlage des EBP verantwortlich gewesen sein könnte.

Der BMI der Patientinnen ist in beiden Gruppen nahezu identisch (26 vs. 27 kg/m^2^) und scheint keinen Einfluss auf die Schwere des PDPH und das sich anschließende Therapieregime zu haben, was sich teilweise mit der Literatur deckt [20], in der aber auch gegenläufige Aussagen zu finden sind [11, 22]. Eine adipositasbedingte Abnahme der PDPH-Schwere scheint sich in der gegenwärtigen Diskussion frühestens ab einem BMI über 31,5 kg/m^2^ einzustellen [22], der aber regelhaft von den Patientinnen in beiden Gruppen deutlich unterschritten wurde.

Eine retrospektive Studie mit 153 Frauen von Dodge et al. untersuchte den Zusammenhang zwischen Nikotinabusus und PDPH und kam zu dem Ergebnis, dass Raucherinnen, verglichen mit Nichtraucherinnen, ein niedrigeres Risiko aufwiesen (13,7 % vs. 34,1 %, *p* = 0,009) [10]. Obwohl der genaue pathophysiologische Mechanismus nicht geklärt ist, nehmen die Autoren an, dass die prokoagulatorische Wirkung vom Rauchen den Verschluss des Duralochs durch Blutgerinnsel unterstützt [10, 16]. Interessanterweise kann man unseren Daten entnehmen, dass sich die Raucherinnen fast ausschließlich in der Non-EBP-Gruppe befanden und damit konservativ behandelt wurden, was einen insgesamt milderen Verlauf des Postpunktionskopfschmerzes vermuten lassen könnte. Dies könnte die oben genannte Hypothese unterstützen.

Möglicherweise ist eine erschwerte PDK-Anlage mit mehreren Punktionsversuchen ein Prädiktor für das Auftreten eines PDPH [29] und könnte daher mit der Notwendigkeit einer invasiven Therapie mittels EBP assoziiert sein. Dies könnte durch ein größeres Duraleck und eine konsekutiv höhere Intensität des PDPH verursacht sein, was durch die Daten unserer Untersuchung bestätigt wird. Die Größe des Duralecks ist dabei vermutlich auch von Typ und Durchmesser der Nadel abhängig. Die deutschen Zentren nutzten für die PDK-Anlage Tuohy-Nadeln in verschiedenen Stärken (16, 17 und 18 G), wobei die 18-G-Nadel mit über 80 % in beiden Gruppen am häufigsten Anwendung fand. Der mögliche Einfluss der Nadelgröße auf die Intensität des Kopfschmerzes kann jedoch durch unsere Studie weder bestätigt noch widerlegt werden.

### Zeitpunkt der EBP-Anlage

Die überwiegende Anzahl von Patientinnen mit einem PDPH wurde mit einem EBP behandelt (56 von 73, Patientinnen, 76,7 %), was im internationalen Vergleich eine vergleichsweise hohe Akzeptanz des EBP als Therapiebestandteil des PDPH vermuten lässt [14].

Patientinnen mit einem PDPH nach ADP sollen ca. 24–48 h konservativ behandelt werden, da die frühe Anlage eines EBP mit einer höheren Versagensrate assoziiert ist [13]. Konservative Behandlungsmaßnahmen beinhalten Bettruhe, Volumengabe, Koffeineinnahme und analgetische Therapie. Unsere Ergebnisse legen nahe, dass die konservativen Maßnahmen die Kopfschmerzsymptomatik bei 76 % der Patientinnen (56 von 73 Patientinnen) nicht ausreichend besserten.

Patientinnen, die einen EBP erhielten, waren mit 85 % medikamentös vorbehandelt, sodass vermutet werden kann, dass die persistierende Kopfschmerzintensität und fehlende Besserung zur Therapieentscheidung für einen EBP beigetragen hat.

Aber wonach richtet sich die Entscheidung für einen epiduralen Blut-Patch? Insgesamt ist das maximale Schmerzniveau der EBP-Gruppe innerhalb der ersten 24 h nach der Diagnosestellung höher als in der Vergleichsgruppe. Das Schmerzniveau nach einer Duraverletzung könnte also Einfluss auf die Wahl der Therapie gehabt haben. Gupta et al. zeigten, dass ein NRS-Wert > 7, die Verwendung einer größeren Nadel (< 18 G) und frontotemporal betonter Kopfschmerz einen Einfluss auf die Entscheidung zum Blut-Patch hatten [13]. Darüber hinaus war der Kopfschmerz bei 10 % der Patientinnen in der EBP-Gruppe so stark, dass die Versorgung des Neugeborenen nicht gewährleistet werden konnte. Auch dies kann die Behandelnden dazu bewogen haben, einer invasiven Therapie zuzustimmen. Der EBP ist die Methode der Wahl für den moderat bis schweren PDPH mit einer Erfolgsrate von 61–98 % laut Literatur [28]. MRT-Studien haben gezeigt, dass sich epidurales Blut an den Durasack anlegt und sich für 18 bis 24 h ein Blutgerinnsel formiert [4]. Da sich das Liquorvolumen erst mit einer gewissen Latenz nach Duraverschluss normalisiert, kann dies allein nicht der Grund für die Schmerzlinderung sein. So wird angenommen, dass die Injektion des Blutes in den Epiduralraum bereits den lumbalen Druck im Subarachnoidalraum erhöht, in dessen Folge der erniedrigte intrakranielle Druck ansteigt und zu einer reflektorischen Vasokonstriktion führt [6].

Der schnelle Effekt des Blut-Patch spiegelt sich ebenfalls in unseren Daten wider: So geben die Betroffenen bereits kurz nach der Anlage einen deutlichen Rückgang des Schmerzniveaus an. Der maximale Schmerz unter Belastung vor dem EBP lag hier bei einem NRS-Wert von 8,5 (7; 10). Demgegenüber steht ein Wert von (0; 2) 4 h nach dem EBP.

Entscheidend für den Nutzen eines EBP könnte der Zeitpunkt der Anlage sein. Von unseren Patientinnen stellten sich 15 von 56 (27 %) der EBP-Gruppe mit anhaltenden Beschwerden erneut stationär vor, welche bei 9 Patientinnen einen zweiten EBP erforderten. Diese 9 Frauen hatten den ersten EBP deutlich früher bekommen als die mit einem Blut-Patch erfolgreich therapierten Frauen (Zeitspanne zwischen ADP und Anlage 1. EBP: 58 vs. 74 h, *p* = n. s.). In der Literatur wird der Cut off zwischen 48 und 72 h gesetzt [18, 21, 24], sodass die „frühe“ Therapie in unserer Gruppe ein Faktor für das Versagen des ersten EBP gewesen sein könnte. Als pathophysiologische Erklärung wird angenommen, dass sich das Blut mit dem initial noch großen Volumen an Liquor im Epiduralraum mischt, was die Blutgerinnung behindert, und kein suffizienter Verschluss des Duralecks erfolgen kann [13].

Die augenblickliche Annahme ist, dass die Symptome eines PDPH nach ADP umso stärker sind, je größer das Liquorleck ist, sodass die Patentinnen mit stärkeren Schmerzen aufgrund des höheren Leidensdrucks früher behandelt wurden. Dies könnte nahelegen, dass die höhere Versagensrate eines „frühen“ EBP eher auf die möglicherweise größere Duraperforation und das größere Liquorleck zurückzuführen ist und nicht zwangsläufig mit dem Zeitpunkt der EBP-Anlage zusammenhängt.

Die Studie von Loeser et al., die einen Benefit eines „späten“ EBP zeigt [19], ist mit der Literatur nur schwer vergleichbar, da der Cut off (früh vs. spät) bei 24 h gesetzt wurde. Es ist also anhand der aktuellen Studienlage noch nicht eindeutig zu empfehlen, die Anlage eines EBP spät zu indizieren, um die Erfolgsrate zu steigern.

### Volumen des EBP

In der Literatur wird das Volumen des injizierten Eigenblutes als Risikofaktor für den Erfolg/das Versagen der Methode diskutiert, wobei das exakte Volumen für einen erfolgreichen epiduralen Blut-Patch nicht bekannt ist. Studien zeigen, dass die Effektivität nicht vom injizierten Volumen abhängig zu sein scheint [21, 25]. Gleiches gilt für unsere Daten, bei denen ein Minimum von 8 ml einem Maximum von 40 ml gegenübersteht, dieses jedoch nicht mit der Schmerzintensität nach einer EBP-Anlage korreliert. Russell et al. verwiesen in ihrer Übersichtsarbeit auf retrospektive Daten von 466 Patienten (davon 394 geburtsmedizinische Patientinnen), die im Mittel 20,5 ± 5,4 ml für einen suffizienten EBP erhielten, ohne dass sich bei höheren Volumina bis 30 ml die Rate an EBP-Wiederholungen geändert hat [23]. Die Autor:innen dieser Arbeit schlussfolgerten, dass aktuell ein Volumen von mindestens 20 ml zur erfolgreichen Behandlung eines PDPH mittels EBP angestrebt werden sollte [23], was auch von anderen Publikationen unterstützt wird [5].

Neben dem Verschluss des Duralecks könnte jedoch auch die Erhöhung des Drucks im Epiduralraum durch die Gabe von Eigenblut zu einer Verminderung des Liquorlecks beigetragen haben, was möglicherweise erklärt, dass eine hohe Spannbreite von Volumina (8–40 ml) effektiv waren.

### Limitationen

Bei den hier vorgestellten Daten handelt es sich um Patientinnen aus den deutschen Zentren der europäischen EPIMAP-Studie. Aufgrund der geringen Fallzahl haben wir bewusst auf eine statistische Analyse verzichtet und die Darstellung der Ergebnisse rein deskriptiv angelegt. Daher sind alle Schlussfolgerungen zurückhaltend formuliert und dienen primär der Hypothesengenerierung. Die Daten wurden aus 9 deutschen Kliniken zusammengeführt, sodass arzt- oder zentrumsabhängige Unterschiede im Patientenmanagement und in der Datendokumentation nicht sicher ausgeschlossen werden können. Weiterhin handelt es sich um eine Kohortenstudie ohne Randomisierung, sodass kausale Zusammenhänge zwischen Schmerzintensität und Therapiemaßnahmen nicht überprüft werden können.

### Zusammenfassung

Der postpunktionelle Kopfschmerz beeinträchtigt das Wohlbefinden junger Mütter stark. Umso wichtiger ist es, die Wahl der Therapie (konservativ vs. EBP) bedacht und mithilfe von erfahrenen Kolleg:innen zu treffen. Potenziell prädiktive Faktoren für einen schweren Verlauf wie mehrfache Punktionsversuche, die Nadelgröße, das Schmerzniveau bei Diagnosestellung sowie patienteneigene Faktoren müssen berücksichtigt werden, ebenso der optimale Zeitpunkt der Anlage und das Volumen des Blut-Patches. Weitere größer angelegte, randomisierte Studien sind notwendig, um den Stellenwert von konservativer vs. invasiver Therapie besser zu definieren.

## Fazit für die Praxis

- Die Schwere des postpunktionalen Kopfschmerzes (PDPH) war in der epiduralen Blut-Patch(EBP)-Gruppe höher als bei den konservativ behandelten Patientinnen.
- Der EBP ist eine effektive Therapie des schweren PDPH (mediane Schmerzreduktion um 6 Punkte auf der NRS).
- Ein Zusammenhang zwischen der Höhe des injizierten Blutvolumens und der Effektivität des EBP wird durch die Ergebnisse dieser Studie nicht bestätigt.
- Bei 27 % der Patientinnen mit einem EBP trat ein stationär behandlungspflichtiges PDPH-Rezidiv auf, 60 % dieser Patientinnen wurden mit einem 2. Blut-Patch behandelt.
- Patientinnen mit einem 2. Blut-Patch hatten den 1. EBP im Durschnitt früher erhalten als Patientinnen mit nur einem EBP (58 vs. 74 h nach akzidenteller Duraperforation, ADP), was die Annahme unterstützt, dass zunächst die konservative Therapie über einen ausreichend langen Zeitraum versucht werden sollte, bevor ein EBP durchgeführt wird.

## Data Availability

Die in dieser Studie erhobenen Datensätze können auf begründete Anfrage beim Korrespondenzautor angefordert werden.
