# Erratum zu: Risikofaktoren und Behandlung des Postpunktionskopfschmerzes – Analyse der deutschen Patientendaten der internationalen EPIMAP-Studie

**DOI:** 10.1007/s00101-025-01552-3

**Published:** 2025-06-17

**Authors:** S. Kroegel, C. von Heymann, A. Schyns-van den Berg, K. Becke, P. Kranke, H. Lewald, S. Müller, E. Muggleton, C. Neumann, H. Ohnesorge, S. Piper, L. Kaufner

**Affiliations:** 1 Klinik für Anästhesiologie und Intensivmedizin CCM/CVK, Campus Virchow-Klinikum, Charité – Universitätsmedizin Berlin, Berlin, Deutschland; 2 Klinik für Anästhesie, Intensivmedizin, Notfallmedizin und Schmerztherapie, Vivantes Klinikum im Friedrichshain, Berlin, Deutschland; 3 Department of Anesthesiology, Albert Schweitzer Hospital, Dordrecht, Niederlande; 4 Department of Anesthesiology, Leiden University Medical Centre, Leiden, Niederlande; 5 Abteilung für Anästhesie und Intensivmedizin, Klinik Hallerwiese-Cnopfsche Kinderklinik, DIAKONEO KdöR, Nürnberg, Deutschland; 6 Klinik und Poliklinik für Anästhesiologie, Intensivmedizin, Notfallmedizin und Schmerztherapie, Universitätsklinikum Würzburg, Würzburg, Deutschland; 7 Klinik für Anästhesie und Intensivmedizin, Klinikum rechts der Isar, München, Deutschland; 8 Klinik für Anästhesie, Notfall- und Schmerzmedizin, Gemeinschaftsklinikum Mittelrhein, Koblenz, Deutschland; 9 Abteilung für Anästhesie, Rotkreuz-Klinikum, München, Deutschland; 10 Klinik für Anästhesiologie und Operative Intensivmedizin, Universitätsklinikum Bonn, Bonn, Deutschland; 11 Klinik für Anästhesiologie und Operative Intensivmedizin, Universitätsklinikum Schleswig-Holstein, Kiel, Deutschland; 12 Institut für Medizinische Informatik und Institut für Biometrie und klinische Epidemiologie, Charité – Universitätsmedizin Berlin, Berlin, Deutschland

**Erratum zu:**

**Anaesthesiologie 2025**

10.1007/s00101-025-01540-7

In diesem Artikel kam es zu einer uneinheitlichen und teilweise fehlerhaften Darstellung der Autor*innenzeile. Einige Vornamen wurden ausgeschrieben, andere lediglich abgekürzt. Zudem wurde ein Vorname vollständig ausgelassen, und in einem Fall war die Schreibweise eines Vornamens fehlerhaft (Sarah-Katharina statt korrekt: Sarah-Catharina). Um eine einheitliche Darstellung sicherzustellen, wurde entschieden, alle ersten Vornamen in abgekürzter Form wiederzugeben.

Der Originalbeitrag wurde entsprechend korrigiert.

